# Prevalence and genetic diversity of rotavirus among children under 5 years of age in China: a meta-analysis

**DOI:** 10.3389/fimmu.2024.1364429

**Published:** 2024-04-16

**Authors:** Yue Li, Sijie Wang, Fan Liang, Sashuang Teng, Fei Wang

**Affiliations:** ^1^ Department of Immunization Program, Hongkou District Center for Disease Control and Prevention, Shanghai, China; ^2^ Shanghai Institute of Major Infectious Disease and Biosafety, and Institutes of Biomedical Sciences, Fudan University, Shanghai, China; ^3^ Key Laboratory of Medical Molecular Virology of MoE&MoH, Shanghai Medical College, Fudan University, Shanghai, China; ^4^ Central Administrative Office, Hongkou District Center for Disease Control and Prevention, Shanghai, China

**Keywords:** pentavalent rotavirus vaccine, rotavirus genotype, meta-analysis, children, China

## Abstract

**Background:**

This meta-analysis was performed to assess the prevalence and circulating strains of rotavirus (RV) among Chinese children under 5 years of age after the implantation of the RV vaccine.

**Material and methods:**

Studies published between 2019 and 2023, focused on RV-based diarrhea among children less than 5 years were systematically reviewed using PubMed, Embase, Web of Science, CNKI, Wanfang and SinoMed Data. We synthesized their findings to examine prevalence and genetic diversity of RV after the RV vaccine implementation using a fixed-effects or random-effects model.

**Results:**

Seventeen studies met the inclusion criteria for this meta-analysis. The overall prevalence of RV was found to be 19.00%. The highest infection rate was noted in children aged 12-23months (25.79%), followed by those aged 24-35 months (23.91%), and 6-11 months (22.08%). The serotype G9 emerged as the most predominant RV genotype, accounting for 85.48% of infections, followed by G2 (7.70%), G8 (5.74%), G1 (4.86%), and G3 (3.21%). The most common P type was P[8], representing 64.02% of RV cases. Among G-P combinations, G9P[8] was the most frequent, responsible for 78.46% of RV infections, succeeded by G8P[8] (31.22%) and G3P[8] (8.11%).

**Conclusion:**

Despite the variation of serotypes observed in China, the G1, G2, G3, G8 and G9 serotypes accounted for most RV strains. The genetic diversity analysis highlights the dynamic nature of RV genotypes, necessitating ongoing surveillance to monitor changes in strain distribution and inform future vaccine strategies.

## Introduction

Rotavirus (RV) infection remains a significant global public health concern, particularly among infants and young children, accounting for substantial morbidity and mortality worldwide (1). In China, the burden of RV-related diseases has been a notable challenge, with RV-associated hospitalizations accounting for 32%–50% of all hospitalizations for diarrhea among infants and children<5 years of age (2, 3).

RVs are known for their genetic diversity, characterized by distinct combinations of G (glycoprotein) and P (protease-sensitive) genotypes. These variations in genotypes have implications for both the severity of illness and vaccine effectiveness, as certain RV genotypes may exhibit differential susceptibility to vaccine-induced immunity.

Vaccination is one of the most effective ways to prevent RV gastroenteritis (RVGE). In 2009, WHO Strategic Advisory Group of Experts recommended the employment of routine RV vaccination in expanded program on immunization (EPI) program for infants worldwide, with a particular emphasis in developing countries with high disease burden (4, 5).

Currently, two RV vaccines have been licensed in mainland of China: the pentavalent RotaTeq (RV5, Merck & Co., USA) and the monovalent RV vaccine (Lanzhou Institute of Biological Products Co., China). RV5, approved by the National Drug Administration on April 12, 2018, is indicated for infants aged 6 to 32 weeks. This vaccine is designed to protect against RVGE caused by serotypes G1, G2, G3, G4, and P1A[8] (6). The monovalent RV vaccine, manufactured by Lanzhou Institute, was introduced in China in 2001.However, the post-vaccination prevalence and genetic diversity of RV among children under 5 years in China continue to be of substantial scientific interest.

To address this, we performed a comprehensive meta-analysis of studies published from 2019 to 2023. This analysis was aimed at monitoring the shifts in RV genotypes in the Chinese pediatric population following the introduction of RV vaccine. Our hypothesis was that the introduction of RV vaccine would lead to a shift in the predominant RV genotypes.

## Materials and methods

### Literature search

We conducted a systematic review and meta-analysis following the Preferred Reporting Items for Systematic Reviews and Meta-Analyses (PRISMA) guidelines (7). We identified studies of diarrhea caused by RV among children less than 5 years old in mainland China, published before November 12, 2023. A comprehensive literature search was conducted in multiple electronic databases, including PubMed, Web of Science, Embase, China National Knowledge Infrastructure (CNKI), Wanfang and Chinese Bio-Medical Literature Service System, China (SinoMed) Data. Keywords and Medical Subject Headings (MeSH) terms related to “rotavirus”, “rotavirus genotypes”, “molecular epidemiology” and “China” were used to identify relevant studies. The search strategy is summarized in the Supplement.

### Review strategy

Endnote X (Thomson Reuters, Inc., Philadelphia, PA) was utilized to create a digital citation library for the articles identified in the database searches. Duplicate records were removed from the combined search results of PubMed, Embase, and Web of Science, using Endnote. Each study was assigned a unique identification code to facilitate tracking through the review and analysis stages. Two independent reviewers initially screened titles and abstracts to determine eligibility, with full-text reviews of potentially suitable studies performed for final inclusion. Any discrepancies between reviewers were resolved through discussion and consensus.

### Study selection criteria

The criteria for including studies in this analysis were as follows: (1) Study Period: Only studies reporting data on RV diarrhea after 2019 were considered. (2) Population: The focus was on children under 5 years old residing in mainland China. (3) Outcome: Studies needed to provide information on the prevalence and genotype distribution of RV. (4) Sample size: Studies included in the analysis had to involve a minimum of 50 strains for G and P typing.

### Data extraction

Data extraction from the chosen studies was performed using a standardized data extraction form. The collected information encompassed study details (such as author, publication year, and study design), particulars about the study population (including sample size and age groups), RV prevalence rates, data on RV genotypes (both G-type and P-type), and any other pertinent variables.

### Quality assessment

The methodological qualities of each article were rigorously evaluated using a modified 12-point scoring system, adapted from Downs and Black’s checklists (8). This evaluation encompassed several key domains: quality of reporting, external validity, and potential biases (including risk and confounding biases), as well as the statistical power of the study. To assign scores, we adhered to a comprehensive quality checklist, which included: a clear articulation of the study objectives; explicit indication of the study design; the representativeness of the sample relative to the target population; participants accrued during the same period; justification of the sample size; effective management of missing data; and thorough exploration and reporting of participant characteristics (age, gender, etc.). This exploration also extended to the reporting of confounding variables, RV detection methods, potential biases, and clearly defined outcomes.

### Data synthesis and analysis

To analyze the temporal and spatial distribution patterns of RV G and P types in China, we examined changes over time and across different regions. The demarcation for these regions was based on the Qinling Mountain-Huaihe River line (9), traditionally regarded as the geographical division between North (warm temperate zone) and South (subtropical zone) China. The northern region of China refers to the north of the line, and the southern region refers to the south of the line. Our stratified analysis focused on disease burden, categorizing data by year (2019-2022), region (northern and southern areas), and age groups (0-6, 11, 23, 35, 47, 59 months). We report the proportion of each RV genotype (P/G), noting that each proportion represents a pooled estimate from studies reporting specific genotypes, with each proportion having its unique denominator. Consequently, these proportions are independent and do not cumulatively total 100%. Prior to data synthesis, we assessed statistical heterogeneity among the included studies using the Cochrane Q and *I* ^2^ statistics (10), considering P<0.1 or *I* ^2^>50% as significant indicators of heterogeneity. In cases of significant heterogeneity, we employed either a random-effects model (11) or a fixed-effects model (12) for pooling estimates. Additionally, we evaluated publication bias using Bgg (13) and Egger’s (14) test, where applicable. All meta-analyses were conducted using STATA software version 12.0 (Stata Corporation, College Station, TX, USA), with a P-value of less than 0.05 denoting statistical significance, unless specified otherwise.

### Ethical considerations

As this meta-analysis utilized data from published studies, ethical approval was not required.

## Results

### Study selection

**Figure 1:**
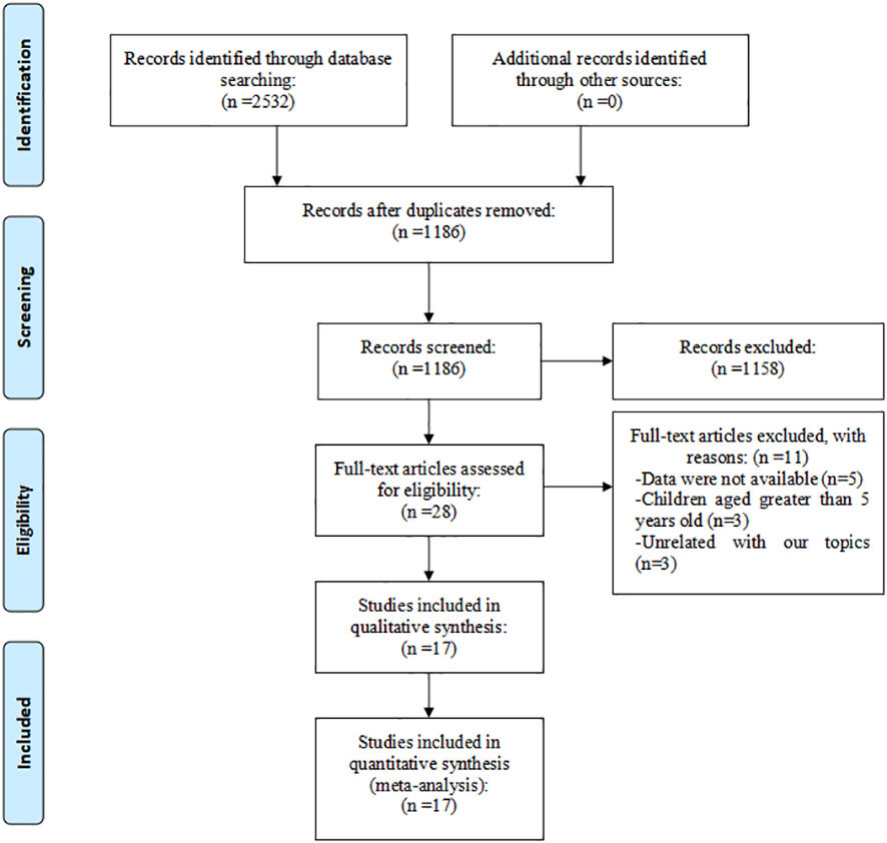
Eligibility of studies for inclusion in meta-analysis.

Our initial search across databases including PubMed, Web of Science, Embase, CNKI, Wanfang, and SinoMed Data yielded 2532 research articles. Of these, 1346 were excluded due to duplication. We then reviewed the titles and abstracts of the remaining 1186 studies, leading to the exclusion of 1158 articles for various reasons. This process resulted in 28 studies being selected for full-text review. However, 11 of these were further excluded due to the following reasons: unavailability of data in 5 studies, inclusion of children older than 5 years in 3 studies, and irrelevance to our research topics in 3 studies. Ultimately, 17 studies (comprising 11 Chinese and 6 English articles) (15–31) were included in our systematic review and meta-analysis (**Figure 1**). Chinese articles refer to articles published in Chinese journals where the language is Chinese. English articles refer to articles published in international journals where the language is English.

### Study characteristics and quality assessment

**Figure 2:**
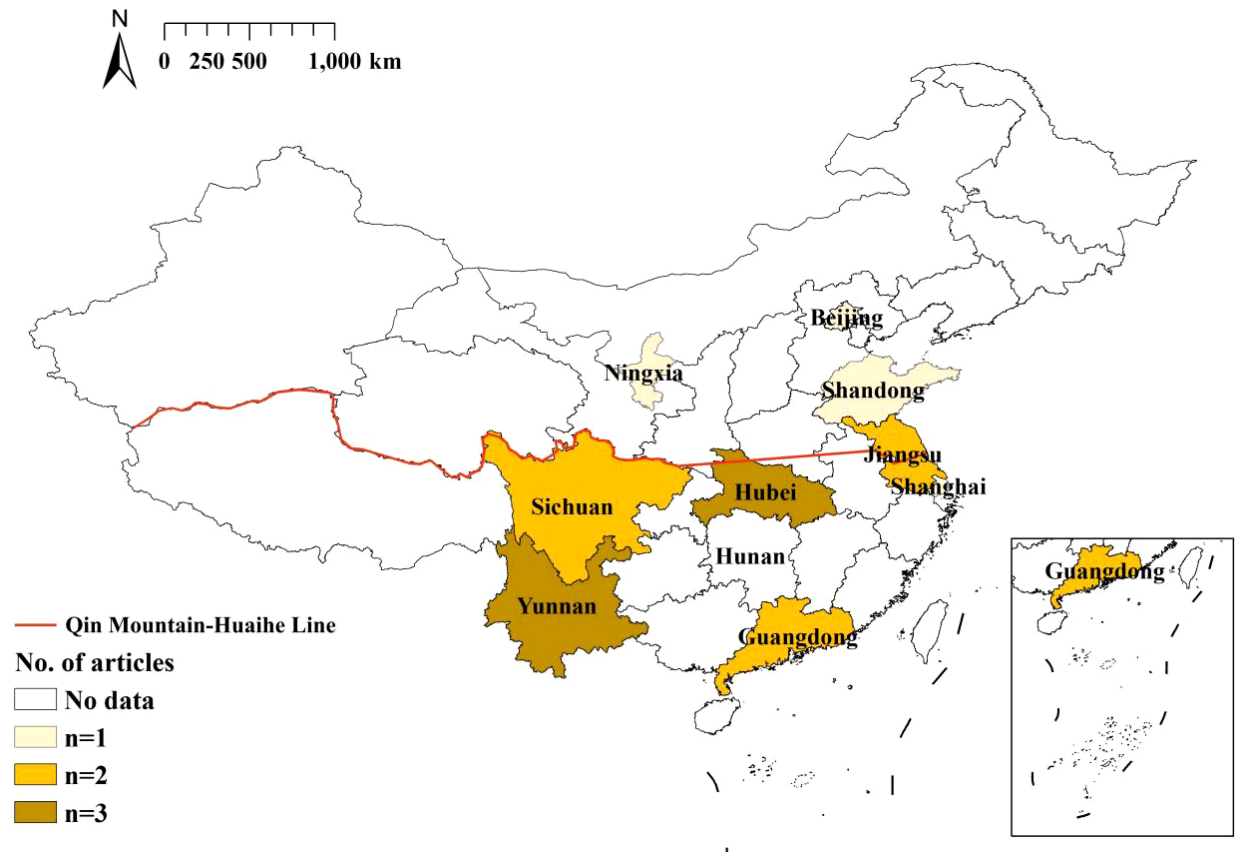
Distribution of included articles by province. China’s Northern region refers to the north of the Qingling Mountain-Huaihe River line, and Southern region refers to the south of the Qingling Mountain-Huaihe River line.

The characteristics and quality assessment scores of these studies are summarized in **Table 1**. The included studies, published between 2019 and 2023, encompassed a wide range of geographical regions within China (**Figure 2**). Notably, all 11 Chinese articles were published in prominent Chinese core domestic journals, highlighting their relatively good quality. Of the 17 studies, a majority, 52.94% (9/17), focused on hospitalized children. A further 29.41% (5/17) of the studies examined outpatient children, and 17.65% (3/17) included both inpatient and outpatient groups. All data were derived from sentinel surveillance systems, ensuring a robust and comprehensive dataset. In terms of diagnostic methods, 76.47% (14/17) of the studies employed the enzyme-linked immunosorbent assay (ELISA) method for detecting RV in diarrheal stool specimens. A slightly higher proportion, 88.24% (15/17), utilized real-time polymerase chain reaction (RT-PCR) for genotyping RV G and P types. The remaining two studies adopted semi-nested or nested RT-PCR methods. Regarding the specific G and P types investigated, the majority of the studies (over 90%) focused on the common G types (G1-G3, G8, G9) and P types (P[4], P[6], P[8]), providing a comprehensive understanding of the predominant strains in the region.

**Table 1:** Baseline characteristics of patients in the studies included in the meta-analysis.

Study	Year	Study site	Study Setting	Design	Duration	Lab Method	Sample Size	No. (%) of RV Positive Cases	Quality Score
Ma XZ (16)	2022	Sichuan Province	Hospitalized	Sentinel surveillance	2019.1-2020.12	ELISA, RT-PCR	365	67(18.4)	A
Zhou SF (17)	2021	Hunan Province	Hospitalized	Sentinel surveillance	2019.1-2020.12	ELISA, RT-PCR	218	82(37.6)	A
Hu JY (18)	2021	Yunnan Province	Outpatients	Sentinel surveillance	2019.1-2019.12	ELISA, RT-PCR	418	132(31.6)	A
Cao YH (19)	2022	Yunnan Province	Hospitalized	Sentinel surveillance	2019.1-2020.12	ELISA, RT-PCR	894	216(24.2)	A
Wang ZH (20)	2022	Jiangsu Province	Hospitalized	Sentinel surveillance	2019.1-2020.12	ELISA, RT-PCR	306	49(16.0)	A
Liu D (21)	2023	Shandong Province	Hospitalized	Sentinel surveillance	2021.1-2021.12	ELISA, RT-PCR	944	47(5.0)	B
Huang XP (22)	2021	Guangdong Province	Hospitalized	Sentinel surveillance	2019.10-2020.9	ELISA, RT-PCR	1512	109(7.2)	A
Li J (23)	2021	Hubei Province	Outpatients	Sentinel surveillance	2019.1-20219.12	RT-PCR	922	116(12.6)	A
Ren D (24)	2021	Sichuan Province	Outpatients	Sentinel surveillance	2019.1-2020.12	ELISA, RT-PCR	378	36(9.5)	A
Kuang XZ (25)	2021	Shanghai	Hospitalized	Sentinel surveillance	2019.1-2020.12	RT-PCR	568	110(19.4)	A
Liang D (26)	2022	Guangdong Province	Hospitalized	Sentinel surveillance	2019.1-2020.1	RT-PCR, Semi-nested PCR	706	103(14.6)	A
Zhou X (27)	2023	Hubei Province	Hospitalized and outpatients	Sentinel surveillance	2019.1-2022.5	RT-PCR	425	94(22.1)	B
Shen S (28)	2022	Jiangsu Province	Hospitalized and outpatients	Sentinel surveillance	2019.1-2019.12	ELISA, RT-PCR	198130	70813(35.7)	A
Jiao Y (29)	2023	Beijing	Outpatients	Sentinel surveillance	2019.1-2022.12	ELISA, RT-PCR	748	82(11.0)	A
Jiang HJ (30)	2023	Yunnan Province	Hospitalized	Sentinel surveillance	2019.1-2021.12	ELISA, RT-PCR	20013	149492(13.4)	A
Zhou X (31)	2020	Hubei Province	Hospitalized and outpatients	Sentinel surveillance	2019.1-2019.12	ELISA, Nested RT-PCR	4409	1125(25.5)	A
Cao M (32)	2023	Ningxia Province	Outpatients	Sentinel surveillance	2019.1-2021.12	RT-qPCR, RT-PCR	2560	660(25.8)	A

RV, rotavirus; ELISA, enzyme-linked immunosorbent assay; RT-PCR, real time polymerase chain reaction; A = 9–12, B = 5–8, C = 1–4.

### Sensitivity analysis

During our data analysis, we observed heterogeneity across most analytical indicators. As a result, we consistently applied the random-effects model throughout the analysis to account for this variability. A comparative evaluation of the outputs derived from all articles, including both Chinese and English publications, versus those obtained solely from English-language articles revealed no significant differences in the temporal and spatial trends of RV strain diversity. However, there were slight inconsistencies in the magnitude of percentages between these two datasets. Given this observation, and to ensure a comprehensive representation of the data, we have chosen to present only the analyses that incorporate all publications, both Chinese and English, in the subsequent sections.

### Rotavirus infection among children under five in China by age, year and gender

**Figure 3:**
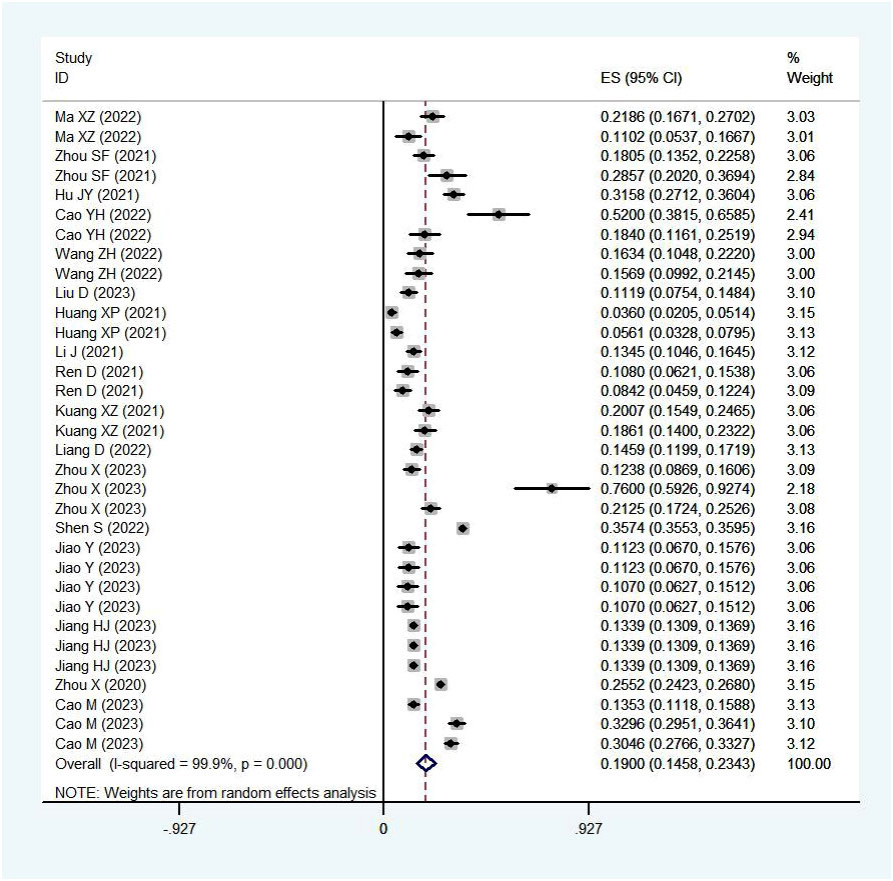
Prevalence of RV infection in children under five.

In our systematic review, RV was detected in 11,330 out of 58,199 stool samples collected from children under five years of age with acute gastroenteritis. The pooled prevalence of RV infection in these children was 19.00% (95%CI: 14.58%-23.43%) (**Figure 3**), with a distribution of 60% males and 40% females.

**Figure 4:**
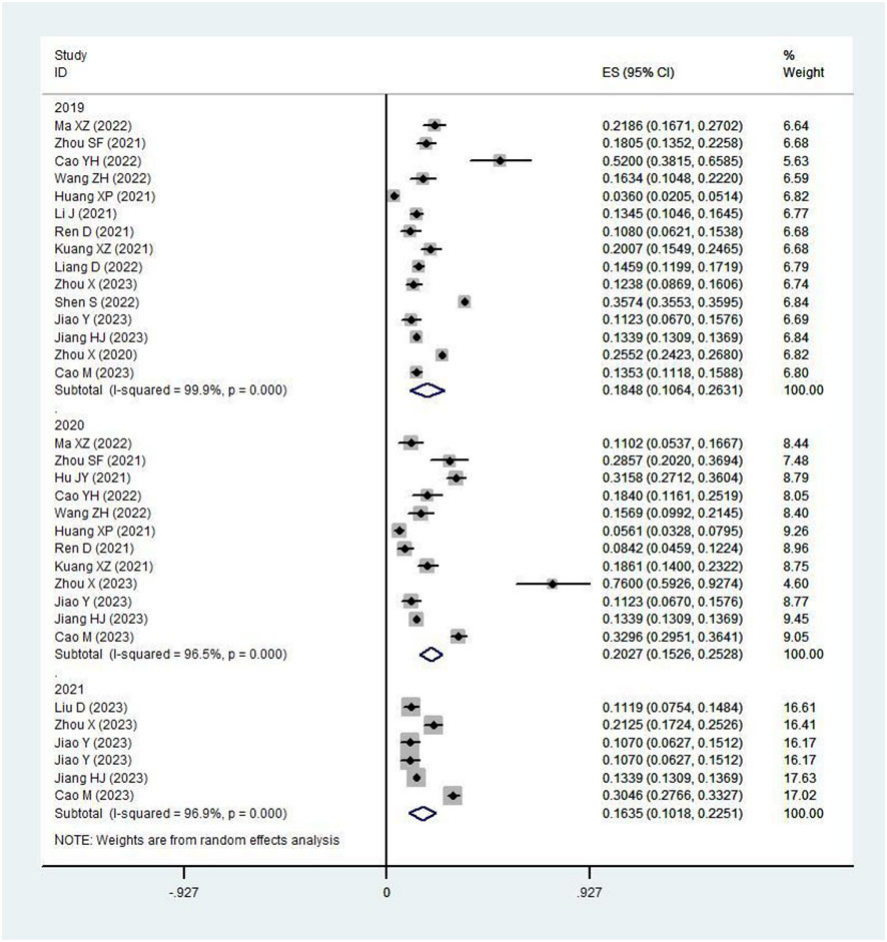
Prevalence of RV infection in children under five by year.

**Figure 5:**
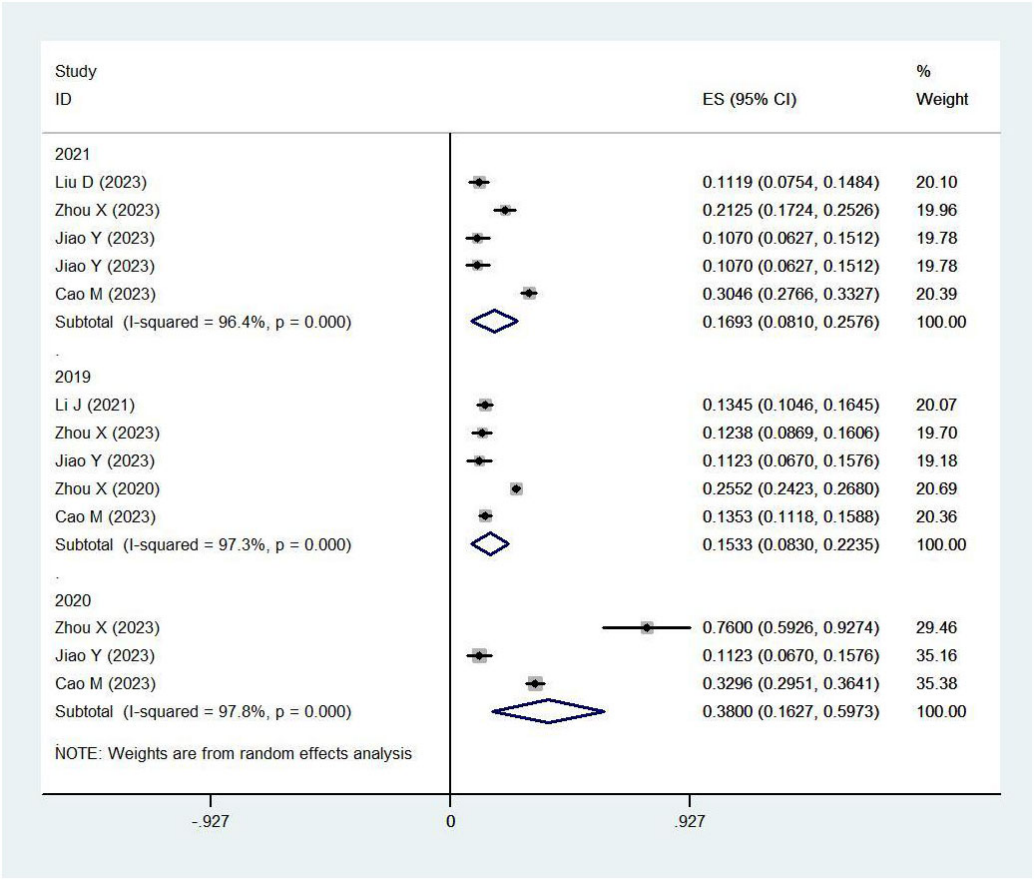
Prevalence of RV infection in children under five in the northern region by year.

**Figure 6:**
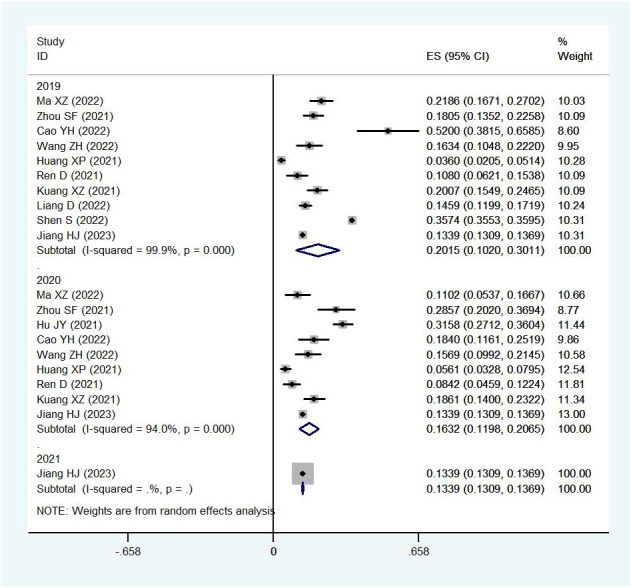
Prevalence of RV infection in children under five in the southern region by year.

Yearly pooled data indicated that the RV prevalence rate was 18.48% in 2019, rose to 20.27% in 2020, and then decreased to 16.35% in 2021(**Figure 4**). When stratifying by region, the Northern region recorded the highest RV prevalence in 2020 (38.00%)(**Figure 5**), while the Southern region’s peak was in 2019 (20.15%) (**Figure 6**; **Table 2**).

**Table 2:** Positive rate of rotaviral diarrhea among children <5 years old in China, 2019-2023, stratified by year, age, care settings and detection methods for RV.

	Pooled positive rate of rotaviral diarrhea among children <5 years old % (95% CI)		
	North % (95%CI)	South% (95%CI)	Overall% (95%CI)
By year			
2019.1-2019.12(n=191,633)	15.3(8.3-22.4)	20.2(10.2-30.1)	18.5(10.6-26.3)
2020.1-2020.12(n=22,958)	38.0(16.3-59.7)	16.3(12.0-20.7)	20.3(15.3-25.3)
2021.1-2021.12(n=18,925)	16.9 (8.1-25.8)	13.4(13.1-13.7)	16.4(10.2-22.5)
By age			
0-5 months(n=54,917)	9.0(0.10-17.9)	15.4(-1.1-31.9)	13.8(0.5-27.1)
6-11 months(n=56,214)	33.3(2.6-64.1)	17.6(1.6-33.5)	22.1(9.6-34.6)
12-23 months(n=61,235)	27.8(23.0-32.7)	24.3(0.3-48.2)	25.8(11.3-40.3)
24-35 months(n=32,349)	18.7(9.9-27.5)	26.7(12.0-41.5)	23.9(12.2-35.7)
36-47 months(n=14,776)	10.2(2.2-18.2)	24.7(16.1-33.3)	18.9(7.9-30.0)
48-59 months(n=14,025)	7.2(1.7-12.7)	19.8(13.1-26.6)	15.1(6.6-23.6)
By care settings			
Outpatients 2019	–	15.0(10.9-19.0)	15.0(10.9-19.0)
Outpatients 2020	–	17.4(12.9-22.0)	17.4(12.9-22.0)
Outpatients 2021	–	13.4(13.1-13.7)	13.4(13.1-13.7)
Hospitalized 2019	21.7(19.6-23.8)	10.8(6.2-15.4)	12.9(11.3-14.5)
Hospitalized 2020	23.0(22.2-27.7)	18.3(15.4-21.2)	21.8(19.8-23.8)
Hospitalized 2021	13.2(11.5-14.9)	–	13.2(11.5-14.9)
Hospitalized and outpatients	28.6(18.8-38.4)	35.7(35.5-36.0)	30.1(21.7-38.5)
By detection methods			
RT-PCR	25.7(17.8-33.6)	17.3(13.7-20.9)	22.8(17.2-28.4)
ELISA and RT-PCR	13.5(5.9-21.1)	18.2(11.9-24.5)	17.0(11.6-22.3)

The age-stratified pooled data revealed the highest RV positive percentage in children aged 12-23 months (25.79%), followed by those aged 24-35 months (23.91%) and 6-11 months (22.08%) (**Table 2**). Interestingly, there was a disparity in the age-related RV prevalence between the Northern and Southern regions. In the North, the highest positive percentage was observed in children aged 6-11 months (33.34%), followed by 12-23 months (27.83%) and 12-35 months (18.73%). Conversely, in the South, children aged 12-35 months had the highest positive percentage (26.73%), with those aged 12-23 months (24.28%) and 36-47 months (24.73%) following closely (**Table 2**).

### Rotavirus positivity in different care settings and different detection methods

Subgroup analysis based on care settings showed that the positive rates of RV in outpatient setting, inpatient setting, and the mixed outpatient+inpatient setting, were 13.43% (95%CI: 12.26%-14.59%), 16.87% (95%CI: 11.11%-22.62%) and 30.08% (95%CI: 21.67%-38.48%), respectively (**Table 2**).

Subgroup analysis based on the method for RV detection showed that, the positive rate of RV was 16.96% (95%CI: 11.63%-22.29%) with both ELISA and RT-PCR methods, and 22.81% (95%CI: 17.20%-28.43%) with the RT-PCR method (**Table 2**).

### Rotavirus genotype distribution

The distribution of prevalent G-types in Chinese children under five years old with RV diarrhea is detailed in **Table 3**. Following the introduction of the RV vaccine, the predominant RV genotype in this age group was G9, accounting for 85.48% of cases. Other genotypes were less common, including G2 (7.70%), G8 (5.74%), G1 (4.86%), and G3 (3.21%) (**Table 3**). Consistently, across various regions, G9 emerged as the most prevalent strain.

**Table 3:** Distribution of common G types among children <5 years old with rotaviral diarrhea in China, 2019-2023.

	Pooled percentage of G types among children with RV diarrhea % (95% CI)					
	G1	G2	G3	G8	G9	G untyped
By Year						
2019(n=3544)	4.9(3.3-6.5)	8.2(-2.4-18.9)	3.2(1.9-4.5)	5.7(-6.7-18.2)	85.5(78.1-92.8)	1.6(0.2-6.3)
2020(n=1013)	0.0(0.0-0.1)	7.7(-6.8-22.2)	0.0(0.0-0.1)	0.0(0.0-0.1)	0.0(0.0-0.1)	11.9(5.2-20.4)
Overall(n=4547)	4.9(3.3-6.5)	7.7(0.0-15.4)	3.2(1.9-4.5)	5.7(-6.7-18.2)	85.5(78.1-92.8)	4.1(2.5-9.5)
By region						
North(n=1085)	0.0(0.0-0.1)	0.0(0.0-0.1)	0.0(0.0-0.1)	13.0(4.0-21.9)	79.6(68.9-90.4)	0.0(0.0-0.1)
South(n=3462)	4.9(3.3-6.5)	7.7(0.6-15.4)	3.2(1.9-4.5)	0.2(-0.2-0.5)	87.9(85.4-90.4)	4.1(2.5-9.5)

China’s Northern region refers to the north of the Qingling Mountain-Huaihe River line, and Southern region refers to the south of the Qingling Mountain-Huaihe River line.

As for the P types, P[8] was the dominant strain, found in 64.02% of cases, followed by P[4] (10.07%) and P[6] (1.85%) (**Table 4**). This P strain distribution was uniform across different years and regions, consistently showing P[8] as the most prevalent strain.

**Table 4:** Distribution of common P types among children <5 years old with rotaviral diarrhea in China, 2019-2023.

	Pooled percentage of P types among children with RV diarrhea % (95% CI)			
	P4	P6	P8	P untyped
By Year				
2019(n=2542)	11.4(-4.3-27.1)	1.9(-1.7-5.5)	57.3(-21.3-135.9)	9.3(-1.3-17.6)
2020(n=326)	7.7(-6.8-22.2)	0.0(0.0-0.1)	84.6(65.0-104.2)	13.6(-0.2-20.5)
Overall(n=2868)	10.1(-0.6-20.8)	1.9(-1.7-5.5)	64.0(-4.1-132.2)	11.3(-0.5-19.7)
By region				
North(n=99)	0.0(0.0-0.1)	0.0(0.0-0.1)	100.0(99.9-100.0)	–
South(n=2769)	10.1(-0.6-20.8)	1.9(-1.7-5.5)	51.7(-6.8-110.2)	11.3(-0.5-19.7)

China’s Northern region refers to the north of the Qingling Mountain-Huaihe River line, and Southern region refers to the south of the Qingling Mountain-Huaihe River line.

In terms of G-P combinations, the most common were G9P[8] (78.46%) and G8P[8] (31.22%). Other notable combinations included G3P[8] (8.11%), G2P[4] (5.02%), and G1P[8] (4.24%) (**Table 5**). The pattern of G-P combinations in the North and South regions mirrored that observed countrywide, with G9P[8], G8P[8], and G3P[8] being the most prevalent strains.

**Table 5:** Distribution of common P types among children <5 years old with rotaviral diarrhea in China, 2019-2023.

G-P combination	Pooled percentage of G-P types %(95% CI)		
	North(n=4145)	South(n=12,007)	Countrywide(n=16,152)
G9P8	76.5(65.9-87.1)	80.1(72.0-88.1)	78.5(72.2-84.8)
G8P8	41.0(9.3-72.6)	5.7(3.8-7.6)	31.2(0.9-61.5)
G3P8	11.5(4.8-18.1)	5.8(3.3-8.3)	8.1(5.2-11.0)
G4P4	0.0(0.0-0.1)	0.9(-0.4-2.2)	0.9(-0.4-2.2)
G1P8	4.6(2.5-6.7)	3.9(1.9-5.8)	4.2(2.9-5.5)
G2P4	4.3(2.5-6.1)	5.2(1.0-9.5)	5.0(2.7-7.4)
G9P4	0.2(-0.2-0.6)	0.6(0.1-1.1)	0.4(0.1-0.2)
G2P8	1.5(-1.4-4.5)	2.0(-1.8-5.8)	1.4(-0.2-2.9)
G1P5	0.0(0.0-0.1)	2.0(-1.8-5.8)	2.0(-1.8-5.8)
G1P4	0.0(0.0-0.1)	0.5(-0.1-1.0)	0.5(-0.1-1.0)
G8P4	0.0(0.0-0.1)	0.2(-0.1-0.5)	0.2(-0.1-0.5)
G4P8	10.7(-3.6-24.9)	1.4(0.4-2.3)	1.4(0.5-2.4)
G3P4	0.0(0.0-0.1)	1.4(0.4-2.3)	1.4(0.4-2.3)
G4P6	0.0(0.0-0.1)	0.2(-0.2-0.5)	0.2(-0.2-0.5)
G3P9	0.2(-0.2-0.6)	0.0(0.0-0.1)	0.2(-0.2-0.6)

### Publication bias

**Figure 7:**
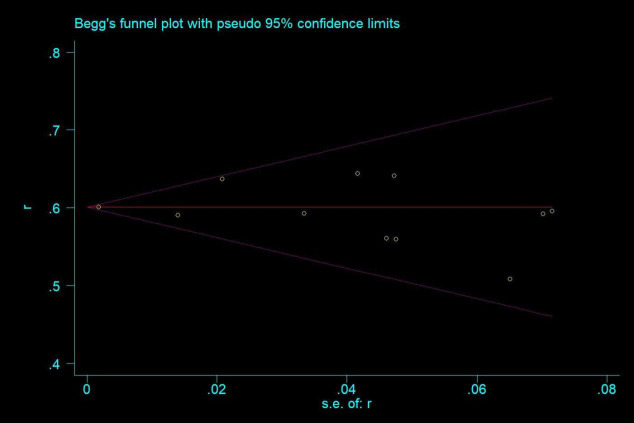
Publication bias assessment.

Publication bias was assessed using Begg and Egger’s test. The results did not indicate significant publication bias among the included studies (Begg’s test: Z=0.78, P=0.436; Egger’s test: t=-0.25, P=0.812) (**Figure 7**).

## Discussion

To the best of our knowledge, this represents the first meta-analytical exploration of RV prevalence and genotypic diversity in the post-RV vaccine era among children under the age of five in China. Our analysis reveals a RV prevalence of 19.00% in this demographic, with a noted predilection towards male children in RV-associated diarrhea cases. A gradation in positivity rates was observed across age groups, with the highest in the 12-23 month age group, followed by 24-35 months and 6-11 months. The serotype G9 emerged as the predominant RV genotype, comprising nearly 90% of infections, while P[8] was the principal P type, accounting for 64.02% of cases. In the context of G-P genotype combinations, G9P[8] was implicated in approximately 80% of RV cases, superseding other combinations such as G8P[8] and G3P[8].

Our study’s finding of a 19.00% prevalence of RV post-RV vaccine implementation contrasts with higher prevalence rates reported in previous studies. A comprehensive analysis encompassing 69 studies between 2011 and 2018 indicated an RV positivity rate of 34.0% (95%CI: 31.3%-36.8%) in inpatient settings and 23.9% (95%CI: 18.1%-30.9%) in outpatient settings, respectively (32). This is higher compared to our findings and even exceeds the 24% RV prevalence rate (95%CI: 22%-26%) observed in a South African meta-analysis of 43 studies conducted over almost four decades (1982-2020) (33). Similar trends of higher RV infection rates have been reported elsewhere. A meta-analysis in Ethiopia, comprising 11 studies from 1981 to 2018, noted a pooled prevalence of 23% (34). In Japan, a 33.0% RV positivity rate was reported three years post-RV vaccine introduction (2014-2015) (35). Additionally, a study in Central Kenya, pre-RV vaccine introduction (2009-2014), detected RV in 27.5% of fecal specimens from children under five with acute gastroenteritis (36). This discrepancy could stem from several factors, including our study period (2019-2023) coinciding with the COVID-19 pandemic and its associated quarantine measures, which likely impacted RV transmission dynamics. Furthermore, varying disease burdens, diagnostic assay sensitivity, and population selection and characterization across studies could contribute to these differences. Additionally, the escalating RV vaccine coverage, as evidenced in a birth cohort study from Shanghai’s Minhang district, might have played a crucial role in diminishing RV prevalence. This study demonstrated a rise in three-dose RV vaccine coverage from 9.0% in the 2013 birth cohort to 29.8% in the 2020 cohort (trend χ² = 37.18, P=0.001) (37).

Our findings underscore a heightened incidence of RV-associated diarrhea predominantly in children aged between 6 and 35 months, with a peak positivity in the 12-23 month age group and a notably lower incidence in infants under 6 months. These findings align with prior meta-analyses conducted in China, encompassing studies from 2011 to 2018 (32), and earlier research before 2011 (38). The earlier study delineated the highest RV positivity rate at 40.1% (95%CI: 33.4%-47.2%) in children aged 12 to 23 months, and the lowest at 17.7% in those aged 0 to 6 months (38). Similarly, the subsequent study reported a peak RV positivity rate of 42.7% (95% CI: 39.3%-46.2%) in the 12-23 month cohort, contrasted with the lowest rate of 17.3% (95% CI: 14.6%-20.1%) in children aged 48-59 months (38). Our results corroborates the WHO recommendation for administering the RV vaccine prior to the age of two, reflecting the age-specific vulnerability to RV infection (39).

In this meta-analysis, the G9 genotype emerged as the predominant strain in RV infections, accounting for approximately 90% of cases. This contrasts with earlier meta-analyses that included studies from China prior to 2019 (32, 40). Our 2014 meta-analysis, incorporating 93 studies published between 1994 and 2012, aimed to evaluate the diversity and temporal shifts in RV strains in Chinese children under 5 years (40). In that analysis, 22,112 and 10,660 RV samples were examined for G and P types, respectively (40). The most frequent G types identified were G1 (39.5%) and G3 (35.6%), with G2 (1.3%) and G9 (0.1%) being less prevalent (40). Another meta-analysis covering 2011-2018, reported G3 (26.1%), G9 (17.5%), and G1 (12.8%) as the most common G types in a similar demographic (32).

These trends suggest a shift in the dominant RV strains over time. For instance, the proportion of G9 escalated from 0.1% during 1994-2012 to 17.5% between 2011-2018, and further to 85.48% in 2019-2020. Conversely, the prevalence of G1 reduced from 39.5% in 1994-2012 to 12.85% in 2011-2018, and then to 4.86% in 2019-2020. Despite these variations, the serotypes G1, G2, G3, G4, and G9 have remained the most common RV strains in recent decades. Notably, our meta-analysis did not identify any G4 strains, as only two studies (22, 29) reported G-P combinations including G4, but without segregating G4 data for separate analysis. Hence, the prevalence of the G4 serotype post-RV vaccine implementation remains unanalyzed.

This genotypic shift in RV prevalence is not unique to China. A similar trend was observed in South Africa, as detailed by Omatola, CA, et al. (41), who analyzed 43 studies from 1982 to 2020 to assess RV infection prevalence and genotypic changes pre- and post-vaccine introduction in children under 5. Pre-vaccine, the predominant G genotypes were G1 (48%), followed by G2 (19%) and G3 (12%). Post-vaccine introduction, G2 (27%) and G9 (25%) emerged as the dominant strains, with G12 (15%) following (41). Comparable shifts towards G2, G9, and G12 genotypes in the post-vaccine era have also been reported in Australia (42) and Zambia (43).

Recent studies have observed an increased prevalence of RV genotypes not represented in vaccine formulations following the integration of RV vaccines into national immunization programs (44, 45). A meta-analysis conducted in South Africa (41) highlighted a significant decline in the G1P[8] genotype (from 43.13% to 11.53%) and a concurrent rise in the G9P[8] (23.22%), G2P[4] (21.2%), and G12P[8] (14.3%) genotypes in the vaccine era. These findings suggest that the introduction of the RV5 vaccine may exert selective pressure on circulating strains, potentially favoring the emergence of less dominant or mutant strains not effectively neutralized by the vaccine. A parallel increase in G2P[4], G9P[8], and G12P[8] genotypes, along with other previously rare genotypes not covered by the monovalent Rotarix vaccine (which targets the G1P[8] strain), has been noted in meta-analyses from Ethiopia and Europe (34, 46).

While some researchers attribute the shifting genotype distribution to inadequate protection against heterologous and newly emerging RV strains, thereby facilitating strain selection due to vaccine-induced immunological pressures (42, 47), others argue that natural fluctuations in strains or gene reassortment events are more likely contributors to the emergence and epidemiological viability of these variants, particularly in contexts of limited herd immunity (48, 49). Despite evidence indicating that the RV vaccine provides both homotypic and heterotypic immunity (50), ongoing national epidemiological surveillance of RV strains in China remains imperative. This is particularly relevant considering the increasing prevalence of the G9 strain, which is not included in RV5, and the observed reduction in genotypes covered by RV5 (G1, G2, G3, and G4) post-implementation. Future research should aim to elucidate the impact of these shifts in RV genotype distribution on the effectiveness of RV5 against RVGE.

This meta-analysis is subject to several limitations. Firstly, the diversity in study design, geographical regions, and sample size among the included studies may contribute to heterogeneity in our results. Additionally, variability in serotyping and genotyping methodologies across these studies could introduce heterogeneity and potentially affect the precision of our findings, albeit less likely the general trends. Secondly, many of the studies predominantly focus on the more common G and P types, specifically G1-G3, G8, G9, P[4], P[6], and P[8]. This focus might lead to misclassification or inability to type less common types like G8 and G12, thereby limiting our comprehensive understanding of the RV strain landscape in mainland China. Thirdly, the analysis encompasses data from studies published between 2019 and 2023. Consequently, ongoing surveillance will be crucial for assessing the long-term variations in RV strains in the post-RV vaccine era.

In conclusion, while this meta-analysis offers an extensive evaluation of RV prevalence and genotypic diversity in Chinese children following the implementation of the RV vaccine, it underscores the necessity for continued national epidemiological monitoring. Such sustained surveillance is pivotal not only for enhancing future vaccine effectiveness but also for understanding the reasons behind any observed vaccine failures.

## Data availability statement

The original contributions presented in the study are included in the article/supplementary material. Further inquiries can be directed to the corresponding author.

## Author contributions

YL: Conceptualization, Data curation, Formal analysis, Funding acquisition, Investigation, Methodology, Project administration, Resources, Software, Supervision, Validation, Visualization, Writing – original draft, Writing – review & editing. SW: Conceptualization, Data curation, Formal analysis, Investigation, Methodology, Software, Supervision, Writing – original draft. FL: Conceptualization, Data curation, Formal analysis, Investigation, Methodology, Supervision, Writing – original draft. ST: Conceptualization, Data curation, Formal analysis, Investigation, Methodology, Project administration, Writing – review & editing. FW: Conceptualization, Data curation, Formal analysis, Investigation, Methodology, Project administration, Software, Supervision, Validation, Writing – original draft, Writing – review & editing.
